# A murine model of ulcerative colitis: induced with sinusitis-derived superantigen and food allergen

**DOI:** 10.1186/1471-230X-5-6

**Published:** 2005-03-03

**Authors:** Ping-Chang Yang, Chang-Sheng Wang, Zi-Yuan An

**Affiliations:** 1 Department of Pathology and Molecular Medicine, McMaster University, Hamilton, Ontario, Canada; 2 Department of Otolaryngology, Shanxi Medical University, the First Hospital, Taiyuan, Shanxi, China; 3 Division of Gastroenterology, Department of Internal Medicine, Shanxi Medical University, the First Hospital, Taiyuan, Shanxi, China

## Abstract

**Background:**

The etiology of ulcerative colitis (UC) is to be understood. The basic pathological feature of UC is intestinal chronic inflammation. Superantigen, such as Staphylococcus enterotoxin B (SEB), is reported to compromise intestinal barrier function by increasing epithelial permeability and initiate inflammation in the intestinal mucosa. Inasmuch as anatomic position of the sinus, chronic sinusitis-derived SEB may follow the secretion and to be swallowed down to the gastrointestinal tract and induce lesions to the intestinal mucosa.

**Methods:**

Sinus wash fluid (SWF, containing SEB) was collected from a group of patients with both chronic sinusitis (CS) and UC. A group of mice were sensitized to ovalbumin (OVA) in the presence of SWF. The sensitized mice were challenged with the specific antigen OVA. The inflammatory status of the colonic tissue was determined with histology, serology and electron microscopy. Using horseradish peroxidase (HRP) as a tracer, another group of mice was stimulated with SWF for 2 hours. The HRP activity was detected in the colonic tissue with enzymatic approaches and electron microscopy.

**Results:**

Epithelial hyperpermeability in colonic epithelium was induced by stimulating with SWF. The HRP activity in the colonic mucosa was almost 11 times more in the SWF treated group (3.2 ± 0.6 μg/g tissue) than the control group (0.3 ± 0.1 μg/g tissue). Mice were sensitized using a mixture of SWF and OVA (serum OVA-specific IgE was detected with a highest titer as 1:64). Challenge with OVA induced extensive inflammation in the colonic mucosa by showing (1) marked degranulation in mast cells (MC, 46.3 ± 4.5%) and eosinophils (Eo, 55.7 ± 4.2%); (2) inflammatory cell infiltration (MC = 145.2 ± 11.4; Eo = 215.8 ± 12.5; mononuclear cell = 258.4 ± 15.3/mm^2 ^tissue); (3) increased MPO activity (12.9 ± 3.2 U/g tissue) and inflammatory scores (1.8 ± 0.3); (4) mucosal surface ulcers; (5) edema in the lamina propria; (6) bacterial translocation and abscess formation in the subepithelial region.

**Conclusion:**

Introducing Sinusitis-derived SEB-containing SWF to the gastrointestinal tract compromised colonic mucosal barrier function increasing epithelial permeability to luminal macromolecular protein in mice. The SWF facilitated colonic mucosal sensitization to luminal antigen. Multiple challenging the sensitized colonic mucosa with specific antigen OVA induced inflammation, induced a condition similar to human ulcerative colitis.

## Background

Ulcerative colitis (UC) is a disease characterized by inflammation and ulcers in the mucosa of the large intestine with unknown etiology. The inflammation usually occurs in the rectum and lower part of the colon, but it may affect the entire colon. The inflammation speeds up colonic motility and causes diarrhea. Ulcers form in places where the inflammation has killed the cells lining the colon; the ulcers bleed and produce pus. Theories about what causes ulcerative colitis abound, but none have been proven. The most popular theory is that the body's immune system reacts to a virus or a bacterium causing ongoing inflammation in the intestinal mucosa; others include genetic predisposition, autoimmune disorders and impaired immune regulation [1-3].

over the past 15 years, more than 2000 patients with chronic sinusitis (CS) including some patients with both CS and UC visited our clinic and were treated with different remedies, including medical treatment and functional sinus endoscopic surgery (FESS). Apart from improvement of chronic sinusitis, those patients with both CS and UC showed great improvement of UC as well (data not shown) that couldn't be explained with the specific treatment alone. Therefore, we postulated that there might be an association between CS and UC in these patients. Microbial infection is the most common cause of CS. The microbial products, such as lipopolysaccharide (LPS), staphylococcus aureus enterotoxin B (SEB) [4,5] can be discharged into the nasal cavity through the natural ostia going backward to the pharynx, and then be swallowed, entering the gastrointestinal tract to affect mucosal physiological functions [6,7].

Staphylococcus enterotoxin B is an extracellular toxin produced by certain strains of *Staphylococcus aureus *(*S. aureus*) [8]. Many cases of food poisoning worldwide involve *S. aureus *enterotoxins [9]. In addition, enterotoxins can be found in cases of toxic shock syndrome [10] and have been implicated in the autoimmune disease rheumatoid arthritis [11]. SEB is synthesized as a precursor protein of 266 amino acids. This precursor is then activated during excretion by cleavage of the N-terminal portion of the molecule. The active enterotoxin B is a single 239 amino acid chain of molecular weight 28,000 daltons and isoelectric point of 8.6 [12]. SEB is a superantigen and possesses powerful immune regulatory capability that results in increased T cell activation and proliferation. SEB-treated Balb/c mice display a dose-dependent colonic inflammation [13]. SEB can also induce colonic epithelial barrier dysfunction [14] that may promote uptake of exogenous antigens, microbial products and other noxious substances into the intestinal tissue to contact immune cells and initiate inappropriate immune reactions.

The nasal cavity and sinus are primary sites of colonization by *S. aureus*, and a quantity of SEB was detected in the nasal cavity of the patients with allergic rhinitis [15,16]. Based on clinical observations, we hypothesized that sinusitis-derived SEB plays a certain role in the pathogenesis of chronic inflammation in the intestinal mucosa via impairing the epithelial barrier function and inducing inappropriate immune reactions. In this study, we aimed to investigate if (1) sinusitis-derived SEB increases intestinal permeability in the mice; (2) the presence of sinusitis-derived SEB facilitates intestinal sensitization to luminal antigen; (3) oral challenge with specific antigen induces intestinal allergic inflammation in the sensitized animals. Accordingly, we developed a murine model of ulcerative colitis with oral allergen in the presence of SEB-containing SWF. The animal model showed intestinal inflammation associated with intestinal mucosal eosinophilia, mastocytosis, acute diarrhea, bacterial translocation and micro-ulcer formation on the surface of the colonic mucosa.

## Methods

### Sinus-wash fluids collected from the patients with CS and UC

Sinus-wash fluids (SWF) were collected from 32 patients with both CS and UC (18 male and 14 female; aged from 26 to 58, with an average of 35.82). We diagnosed CS in the patients as an inflammation of the sinus mucosa with a persistent mucoid or mucopurulent nasal discharge for longer than 3 months that resisted antimicrobial therapy and antral irrigation. Diagnosis was made on the basis of clinical history, rhinoscopic findings, and computed tomographic scan of paranasal sinuses. CS was confirmed by computed tomographic examination that showed diffuse mucosal thickening in the ethmoid or/and maxillary sinuses bilaterally with scores higher than 12 by the Lund-Mackay staging system [17]. Twenty-five healthy medical students were enrolled in this study, their nasal wash fluids were used as controls.

The diagnosis of UC was based on clinical history, colonoscopy and histology. The history included persistent bloody diarrhea, rectal urgency, or tenesmus. Examinations and sigmoidoscopy and biopsy were performed to confirm the presence of colitis and to exclude the presence of infectious etiologies.

Every patient underwent maxillary sinus puncturing and washing. The SWF was collected prior to other procedures. Five ml saline was injected into the sinus cavities and re-collected and stored at -70°C for further use. SEB content in SWF was evaluated with ELISA (All the reagents used in this study were purchased from Sigma unless otherwise mentioned). None of the subjects had recent upper respiratory acute infections. This study was approved by the Ethical Committee of the First Hospital of Shanxi Medical University.

### Animals

For the purpose of verifying our hypothesis of an association between sinus pathology and colitis, an animal model of ulcerative colitis was developed. Mice were sensitized to a model food antigen, ovalbumin (OVA) with or without the presence of SEB-containing SWF. Animal experiments were approved by Animal Use and Care Committee at Shanxi Medical University. Male Balb/c mice (10 week old) were purchased from Beijing Animal Research Institute and maintained in the animal center at Shanxi Medical University. All mice were housed according to guidelines of the animal center. Water was available continuously through automatic ports, and a commercial mouse diet was provided ad libitum.

### Effect of SEB containing SWF on colonic epithelial permeability in the mice

A group of mice was introduced 0.2 ml SWF (containing SEB 50 μg and 10 mg horseradish peroxidase, HRP. SWF was concentrated with the method of ammonium sulfate precipitation for higher content of SEB) via intragastric gavage under a light anesthetization. Mice were killed by cervical dislocation 2 hours later (based on preliminary results; data not shown). Control groups were designed as: a naïve control group, treated with HRP 10 mg in 0.2 ml PBS via intragastric gavage; an inactivated-SWF control group, the SWF was pretreated with anti-SEB (100 μg in 0.2 ml) for 30 min, then introduced to the mice in gavage with HRP. Colon was removed; a piece of colon (3 cm) was opened; the contents were collected and dissolved in 1 ml PBS for HRP assay; the colon tissue was snap frozen and stored at -70°C for HRP assay; another piece of colon (2 × 2 × 4 mm) was fixed with 2.5% glutaraldehyde for 2 hours; then rinsed in sodium cacodylate buffer, incubated in 3,3'-diaminobenzidine tetrahydrochlorine and H_2_O_2 _(pH 7.6) for 30 min, and postfixed with 2% osmium tetroxide for 60 minutes, followed by staining en bloc with 4% uranyl acetate for 30 minutes. Tissue samples were dehydrated through a graded series of ethanol, cleared in propylene oxide, and embedded in Epoxy embedding medium. Thin sections were prepared and stained with 4% uranyl acetate for 5 min and subsequently with 2.5% lead citrate for 2 min and examined at 80 kV with a JEM 1200 electron microscope. HRP containing endosomes were photographed randomly for further analysis.

Frozen colon tissue was weighed and immersed into lysis buffer (20 mM Tris-HCl (pH 7.5), 150 mM NaCl, 1 mM Na_2_EDTA, 1 mM EGTA, 1% Triton, 2.5 mM sodium pyrophosphate, 1 mM β-glycerophosphate, 1 mM Na_3_VO_4 _(sodium ortho-vanadate), 1 μg/ml leupeptin) and homogenized on ice; the homogenates and colon content were centrifuged at 1,500 g for 20 minutes at 4°C respectively, the supernatants were collected for HRP assay. HRP amount in colon content and colon tissue was determined by assaying enzyme activity in the collected supernatant according to the previous report [18]. Briefly, a 0.2-ml sample of supernatant was mixed with 2.8 ml phosphate buffer (0.1 M, pH 6.0) containing 0.003% H_2_O_2 _and 0.025 ml of a solution of o-dianisidine di-HCl (10 mg/ml). The optical density was determined at 460 nm with a spectrophotometer.

### Intestinal sensitization to luminal OVA with the presence of SEB-containing SWF

Ten mice were used for each group. A group of mice was sensitized by means of intragastric gavage with 50 μg OVA in 0.2 ml alum-SWF (the adjuvant, prepared with SWF instead of distilled water that contains 50 μg SEB). Another group was also treated with the same protocol, but the SWF in the adjuvant was pretreated with an anti-SEB antibody (100 μg in 0.2 ml). Control groups were designed as: mice were only exposed to OVA without the presence of SWF, or were only exposed to SWF without OVA; naïve controls were treated with 0.2 ml saline. Each mouse was injected with 50 ng pertussis toxin peritoneally.

### Mice were challenged with specific antigen OVA

From the 14th day after sensitization, all mice were challenged with 50 mg OVA in 0.3 ml saline by means of intragastric gavage 3 times, 48 h apart. Diarrhea was determined by visually monitoring mice for up to 1 hour following intragastric challenge. Mice demonstrating profuse liquid stool were recorded as diarrhea-positive animals. Two days after the last challenge, mice were sacrificed by decapitation; blood samples were collected, serum was separated and stored at -70°C for further analysis. Colon was removed, one piece was fixed with 4% paraformaldehyde for histology; one piece was snap frozen for MPO analysis; one piece was fixed with Carnoy solution for mast cell count; one piece was fixed with 2% glutaraldehyde for electron microscopy.

### Myeloperoxidase activity evaluation

Myeloperoxidase (MPO) activity was determined according to the method of Bradley et al [19]. Tissue samples were homogenized in hexadecyltrimethylammonium bromide buffer in a glass homogenizer on ice. The homogenates were centrifuged and MPO activity in the supernatants was determined. One unit of MPO activity was defined as the amount required to degrade 1 mM H_2_O_2 _in 1 minute at 25°C.

### Colonic mucosal inflammatory score

The morphology of the epithelium, villi, and subepithelial layer of colon mucosa were assessed, and the numbers of eosinophils and mononuclear cells were counted in 10 randomly selected fields (magnification, ×400) for each mouse (100/group). To determine mast cell numbers, tissues were fixed in Carnoy's fixative and paraffin sections were stained with 0.5% toluidine blue. Mast cells were counted in 10 fields for each mouse (100/group). Cell numbers were expressed per mm^2 ^of mucosa. All sections were coded to avoid observer bias. The degree of inflammation on microscopic tissue sections was scored as follows: (from 0 to 4, 0 indicates normal, 4 indicates severe condition): 0, no leukocyte infiltration; 1, low level of leukocyte infiltration; 2, moderate level of leukocyte infiltration; 3, high vascular density and thickening of the colon wall; and 4, transmural leukocyte infiltration, loss of goblet cells, high vascular density, and thickening of the colon wall. Grading was done in a blinded manner [20].

### Observation of mast cell and eosinophil activation

Colon tissue was processed with routine procedures and observed with an electron microscope. Activation of mast cell and eosinophil in colonic mucosa was determined by the phenomenon of degranulation [21].

### Serum specific IgE measurement

Passive cutaneous anaphylaxis (PCA) was employed to determine serum OVA-specific IgE level. The serum was diluted in phosphate-buffered saline (PBS) from 1:4 to 1:128. The mice were injected intradermally with 50 μl serum into each of 4 dorsal shaved skin sites. The sites were outlined with a water-insoluble red marker. Forty-eight hours later each mouse received an injection of 0.1 ml OVA (1 mg/ml) containing 4% Evans blue via the tail vein. The results were recorded thirty minutes after the challenge; the diameter of the blue spot on the injection sites larger than 6 mm was recorded as positive reaction.

### Statistics

Data were expressed as mean ± SD; Student *t *test was used to compare the difference between groups. p < 0.05 was accepted as significant criteria.

## Results

### Staphylococcus enterotoxin B was detected in SWF from the patients with both CS and UC

The content of SEB was significantly higher in the SWF of patients with both UC and CS (from 30.5 to 565.6, with an average of 154.5 ± 81.7 pg/ml) than in the nasal wash fluids (from 0 to 18.6, with an average of 8.5 ± 4.3 pg/ml) of healthy control subjects.

### Stimulation with SWF increased mouse colonic mucosal permeability to luminal macromolecular protein HRP

**Figure 1:**
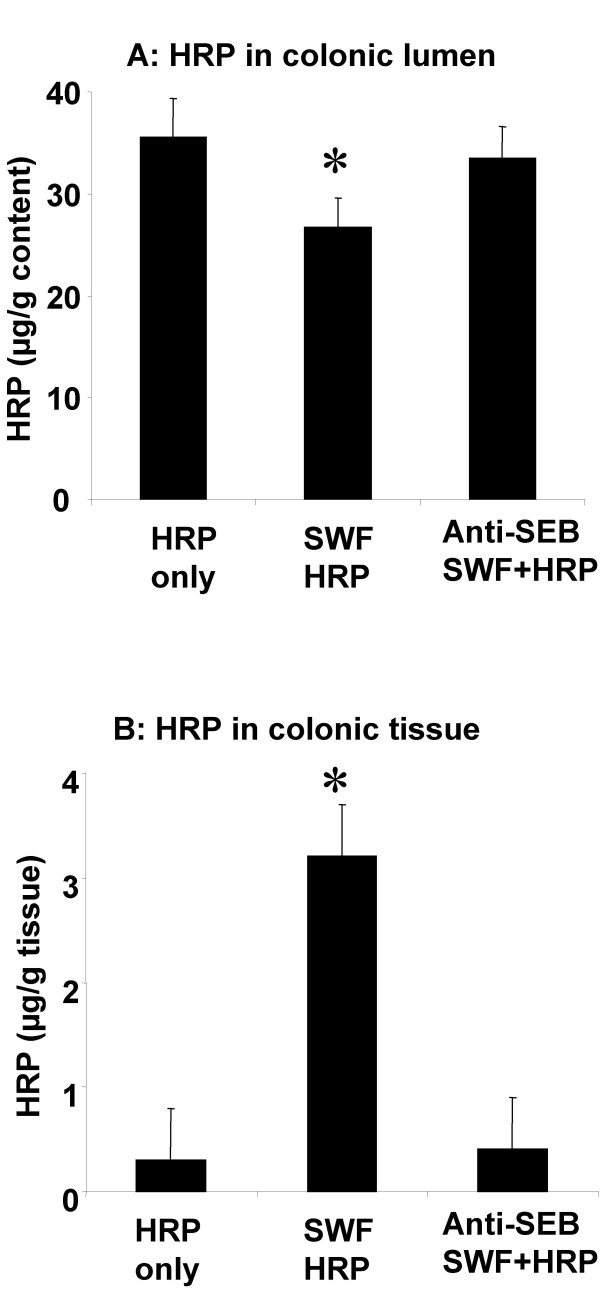
**Mouse colonic epithelial permeability increased by stimulation of SWF**. Bars stand for HRP activity in colonic content (Fig 1A) and colonic tissue (Fig 1B). Each group consists of 10 mice. *, p < 0.05, compared with controls.

**Figure 2:**
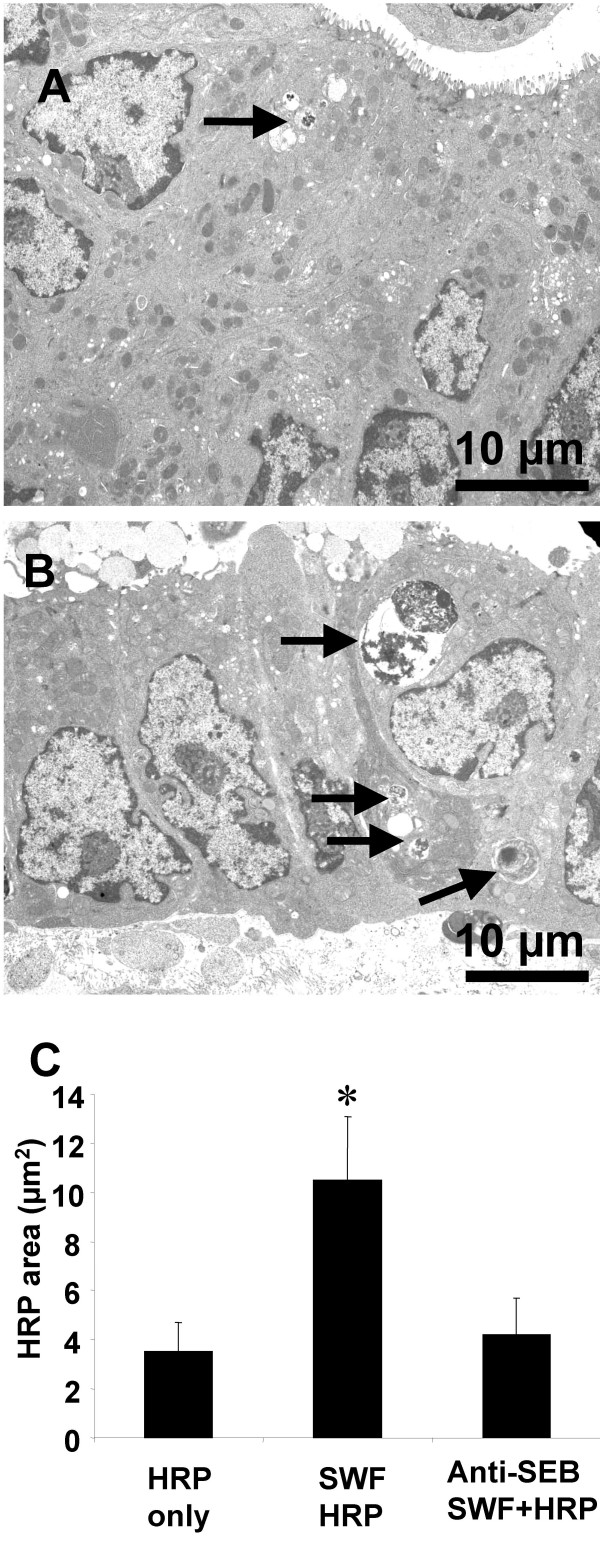
**HRP endosomes in the colonic mucosa**. Representative EM photomicrographs were taken from colonic mucosa of control (Fig 2A) and sensitized mice (Fig 2B). Bars stand for HRP endosome area in 300 μm^2 ^cell area (Fig 2C). *, p < 0.05, compared with controls.

Horseradish peroxisase activity was detected in colonic content in both control mice (group of HRP-only and group of anti-SEB treated SWF) and SWF treated mice (Fig 1A). The HRP activity was significantly higher in the colonic tissue of the mice treated with SWF compared to controls (Fig 1B). Observation with EM revealed that few HRP endosomes of small size were observed in the upper region (above the nucleus) of cytoplasm in the colonic epithelium of control mice. Large size HRP endosomes were observed in both upper and lower (below the upper edge of the nucleus) regions of the mice treated SWF. Image analysis of HRP endosome showed that the HRP endosome area was significantly larger in the mice treated with SWF comparing with those control mice (Fig 2).

### Serum specific anti-OVA IgE was detected in the mice treated with SWF and luminal OVA

Passive cutaneous anaphylaxis (PCA) is the gold standard method to measure allergen-specific IgE antibody levels in mouse models of allergy. The present study demonstrated that only the group treated with both SWF and OVA showed positive results with the highest titer 1:64. No specific IgE was detected in the serum of mice in control groups.

### Activation of mast cells and eosinophils in the colonic mucosa of the sensitized mice after challenging with specific antigen

**Figure 3:**
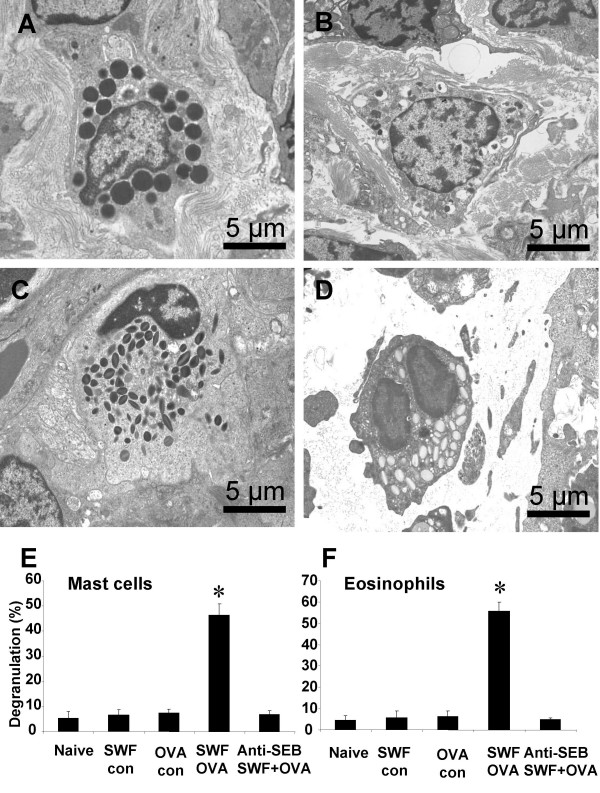
**Activation of mast cell and eosinophil in the colonic mucosa**. Representative photomicrographs (10 for each mouse) were taken from colonic mucosa of naïve mice (Fig 3A, 3C) and sensitized mice (Fig 3B, 3D). Bars stand for ratio of degranulation that was calculated with the numbers of degranulated granules divided by the numbers of total granules of mast cells (Fig 3E) and eosinophils (Fig 3F). *, p < 0.05, compared with naïve controls.

Activation of mast cells and eosinophils was observed as degranulation of the granules in the cytoplasm. Piecemeal type degranulation was observed more often than the anaphylactic type in the mast cells in the colonic mucosa of the sensitized mice with EM. Crystal core degranulation and matrix degranulation were observed in eosinophils of the sensitized mice. Granules were categorized intact or degranulated for each mast cell or eosinophil. Each type granule was numerated with EM. After challenge with specific antigen OVA, degranulation was observed in both mast cells and eosinophils of the sensitized mice. The ratio of degranulation was much higher in the sensitized mice compared with controls (Fig 3).

### Histopathology of colonic mucosa of the sensitized mice after challenge with specific antigen

**Figure 4:**
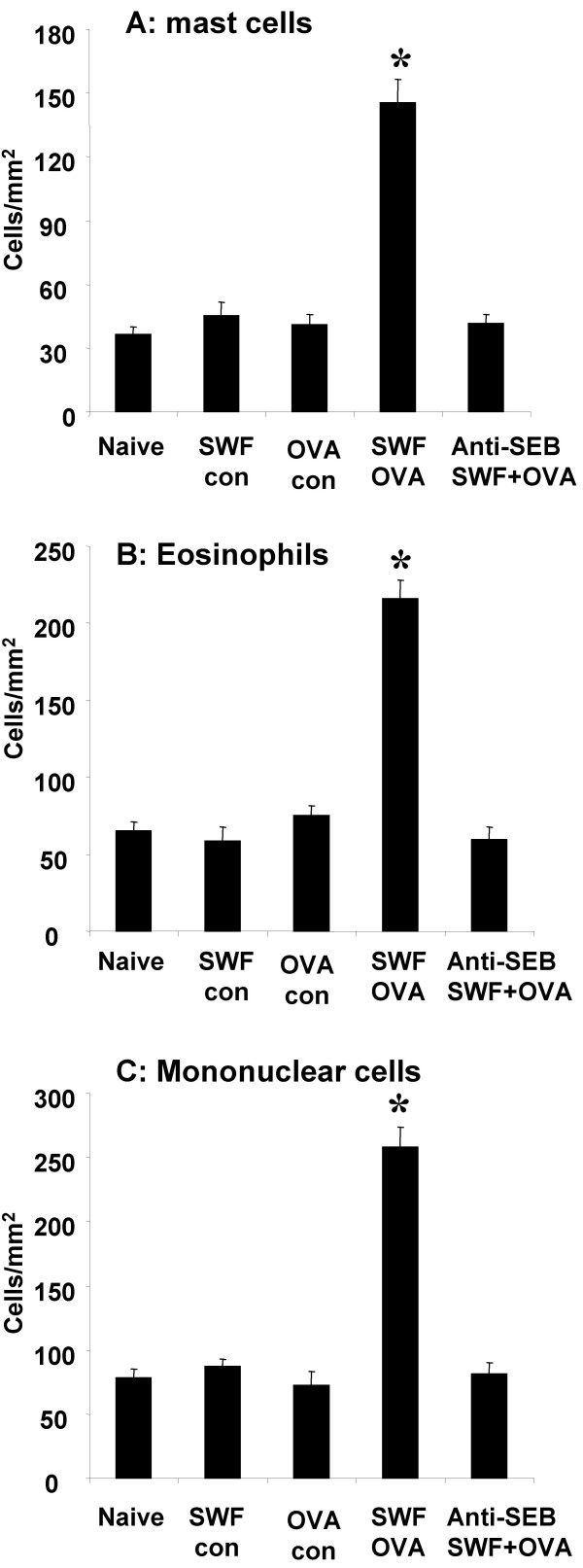
**Inflammatory cell infiltration in the colonic mucosa. **Bars stand for numbers of mast cell (Fig 4A), eosinophil (Fig 4B) and mononuclear cell (Fig 4C). Cell numbers are expressed as cells/mm^2 ^tissue. *, p < 0.05, compared with naïve controls.

**Figure 6:**
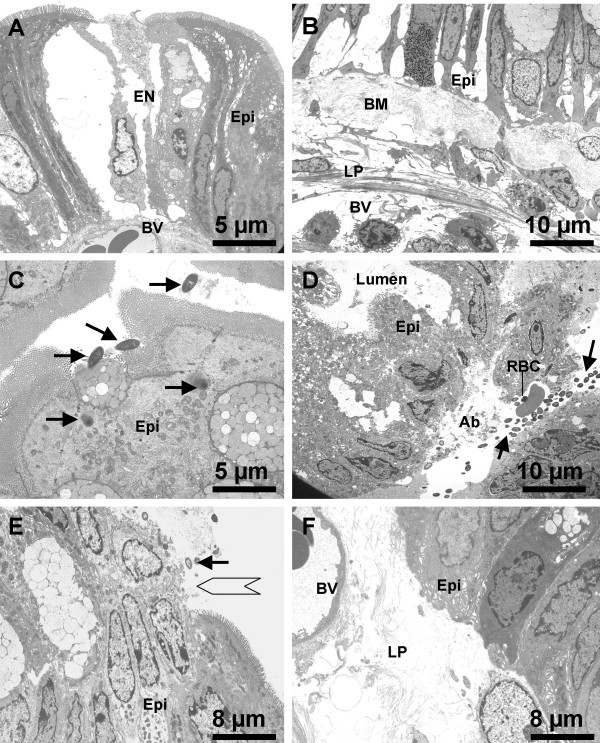
**Ultrapathology of the colonic mucosa of the sensitized mice after challenge with OVA. **Representative EM photomicrographs are taken from the colonic mucosa of the sensitized mice after challenge with OVA and show (A) epithelial cell (epi) necrosis (EN) and dilation of the blood vessel (BV) in the subepithelial region (×3,000); (B) epithelium destruction, basement membrane (BM) hyperplasia and blood vessel dilation (×2,000); (C) bacteria (arrows) adhering to and penetrating the epithelial cells (×3,000); (D) abscess (Ab) formation in subepithelial region with a colony of bacteria (arrows) and a red blood cell (RBC) in it (×2,000); (E) micro-ulcer (empty arrow) formation on the surface of colonic mucosa with bacteria (arrow) adherence (×2,500); (F) edema and blood vessel (BV) dilation in the lamina propria (LP) (×2,500).

Challenge with specific antigen OVA resulted in inflammatory cell infiltration in the lamina propria and subepithelial regions. The infiltrate consisted of mainly mast cells, eosinophils and mononuclear cells. Compared with control, the sensitized group showed significantly more inflammatory cell infiltration, most of them might be activated immune cells (Fig 4). Capillary or small vein dilation was observed in the subepithelial region of the intestinal mucosa after OVA challenge; profound edema in the tissue of the same area was frequently observed (Fig 6B, 6F).

**Figure 5:**
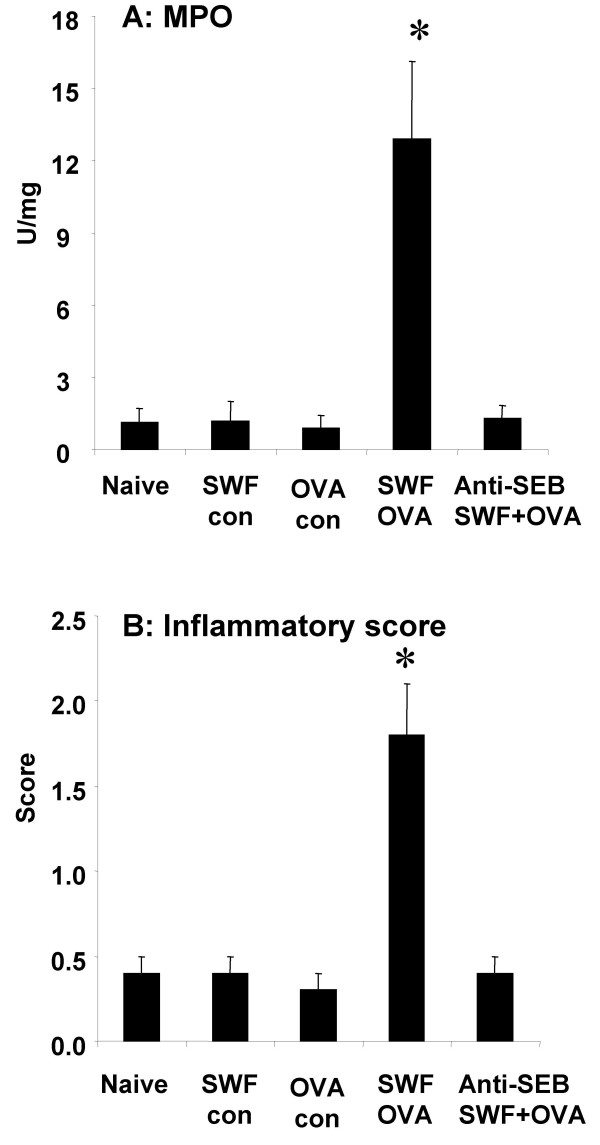
**MPO activity and inflammatory scores of the colonic mucosa. **Colonic tissues show an increased MPO activity (A) and increased inflammatory scores (B) after challenge with OVA. *, p < 0.05, compared with naïve controls.

Inflammatory status in the colonic mucosa was assessed by colonic tissue MPO measurement, inflammatory cell infiltration and mucosal surface condition observation. MPO is a critical parameter that indicates activation of neutrophils. We observed a significant increase in MPO and inflammatory score in colonic mucosa after challenge with OVA in the mice treated with SWF and OVA (Fig 5).

Electron microscopy revealed ultrapathology in the sensitized and OVA-challenged colonic mucosa that included: (i) epithelial cell destruction (Fig 6A, B); (ii) bacteria adherent to or penetrating the epithelial cells; (iii) bacteria translocated to the subepithelial region and abscess formation (Fig 6C, D); (iv) micro-ulcer formation on the surface of the colonic mucosa (Fig 6E); (v) edema in lamina propria (Fig 6F).

### Challenge with specific antigen OVA induced diarrhea in the sensitized mice

Following the protocol of challenge with specific antigen OVA, the sensitized mice developed diarrhea 15–30 minutes after each challenge that lasted up to 1 hour. The number of diarrhea episodes (3 to 7 times) increased in parallel with the number of OVA challenges. No diarrhea was noted in control mice although those mice also received the same number of OVA challenges. Diarrhea was also noted by direct observation of the colon and cecum; the liquid stool observed following OVA challenge-induced diarrhea in the sensitized mice contrasts with the solid pellets seen in the distal colon of other mice in control groups.

## Discussion

Our knowledge about the etiology of ulcerative colitis is still limited. Although some theories about its origins have been advanced, such as genetic predisposition, autoimmune disorders, infection, and so on [23,24], the precise pathogenesis needs to be further understood. In clinical practice, we noted a close association between CS and UC in some patients and their UC was significantly improved after having removed sinus pathology (data not shown). The results of animal experiments verified our speculation: superantigen SEB from sinusitis cooperated with ingested antigen to induce intestinal sensitization. Challenge with the obligate antigen initiated colonic mucosal inflammation as well as the clinical symptom diarrhea. Book DT et al [25] also noted the same phenomenon and suggested that IBD was more prevalent in those people with chronic sinusitis than in other populations.

Rhinosinuses are empty cavities lined with mucosa. The anatomic feature, only having a small ostium, makes them very easily to be blocked and subsequently infected. Infection with *S. aureus *in sinuses is frequently encountered [4,5]. Thus, chronically infected sinuses may be a source of SEB that is released to nasal cavity frequently. A mucus blanket on the surface of nasal mucosa naturally traps small particles from air and the secretions from sinuses and removes them subsequently. Since the direction of the locomotion of the mucus blanket is backward, people sometimes swallow the secretions into the gastrointestinal tract (e.g., during sleep).

There are many toxic substances in the secretions from chronic sinusitis. SEB is one that has been well characterized. The unique feature of SEB is that it can down regulate intestinal barrier function [6,13], activate T lymphocytes without the help from antigen presenting cells to activate T cells. Superantigens bind directly to MHC class II molecules and to a subset of T-cell receptor (TCR) Vβ chains [26,27]. Unlike conventional antigens, superantigens do not require processing by antigen-presenting cells to activate immune cells [31]. Administration of superantigen results in initial selective expansion of T cells that bear specific Vβ chains that recognize the superantigen, followed by their deletion [29]. Another unique feature of superantigen is that it mutes T suppression cell function and promotes Th1/Th2 skewing [30]. It primes an environment to develop sensitization in local tissue. The results in the present animal experiments are consistent with previous studies. Mice treated with SEB-containing SWF and OVA developed intestinal sensitization, but not in those mice treated with only OVA, or SEB-depleted SWF plus OVA. This finding demonstrates that SEB plays a crucial role in the sensitization of the intestinal mucosa to luminal antigen in these mice. Louini D et al [31] reported that SEB also directly sensitized skin and caused Th2 pattern inflammation in the local skin.

Intestinal epithelial cells form a barrier between the luminal contents and the subepithelial region. The barrier restricts substances to be absorbed. It only allows some small molecules such as water to pass it freely. Antigens are macromolecular proteins that are not allowed to be absorbed before being digested to small peptides or amino acids under normal physiological conditions. But in reality, intact antigens do pass the intestinal barrier to reach lamina propria to induce inappropriate immune reactions under certain circumstance. How antigens cross the intestinal epithelial barrier is still a mystery. By introducing both SWF and HRP to mouse gastrointestinal tract, intact HRP in the colonic tissue was increased nearly 11 times compared to control. The results implicate that superantigen SEB is one of the factors that facilitate antigens to be absorbed without destroying their antigenicity. Lu et al also reported that SEB significantly increased colonic mucosal permeability in a mouse study [13].

We have begun to appreciate that food allergy plays a role in the inflammation of intestinal mucosa [32]. The results of this animal model support that inappropriate immune reactions initiate intestinal inflammation. Simply delivering OVA to gastrointestinal tract did not show sensitization in the mice while the combination of SEB-containing SWF and OVA induced sensitization in the colonic mucosa. Based on these data, we suggest that SEB facilitate sensitization of the intestinal mucosa to OVA. The mechanism behind this phenomenon might be that SEB increases permeability of the intestinal mucosa [6,13]. Thus OVA in the intestinal lumen can be transported to deep region of the mucosa. This exogenous protein then contacts the local immune cells to initiate inappropriate immune reactions and sensitizes the mucosa subsequently. The results of challenge with OVA show extensive inflammation in the colonic mucosa in this study. The inflammatory changes may be a result of local mast cell degranulation in response to OVA challenge. Mast cells release chemical mediators such as histamine that is able to increase vascular permeability and to induce edema in the tissue that was noted in the present study and also reported elsewhere [33].

In chronic allergic diseases such as asthma, during continuous antigen exposure, eosinophils are primed by IL-5 and attracted by chemokines, infiltrating the local tissue [34]. We also observed extensive eosinophil infiltration in the colonic tissue after three challenges with specific antigen OVA in the present study. These eosinophils are believed to be responsible for the late phase of the allergic reaction, producing the major basic protein which is toxic to the epithelium [34]. The micro-ulcers on the surface of colonic mucosa may be caused by eosinophil activation. Supportive evidence acquired from EM observation demonstrates that most eosinophils have been activated by showing extensive degranulation. We also noted this phenomenon in the jejunal mucosa in the late phase reaction in a rat model of food allergy [32].

Marked mast cell hyperplasia in the colonic tissue was observed in this study. In general, mast cell numbers in tissues are relatively constant, even though mast cell hyperplasia is observed in both the inflammatory and in repair/remodeling stage of various inflammatory disorders [35]. The functional significance of the accumulation of mast cells in these processes is largely unknown. In allergy, apart from their classical role in eliciting the early phase, mast cells also have an important role in late and chronic stages as we observed in a previous study [32]. In these stages they may interact with and be activated by infiltrated inflammatory cells and by resident structural cells such as epithelial cells, smooth muscle cells and fibroblasts. In the case of allergic reaction, mast cells are mainly activated by the mechanism of IgE mediated FcεRI bridging that accounts for the mast cell activation in the present study. The roles of mast cells in the late phase reactions may be amplified by eosinophils, platelets and neutrophils [36]. If a sensitized patient frequently ingests an obligate antigen unconsciously whereas the allergic reaction only reaches subclinical level, an early inflammation may progress without being noticed until reaching the advanced stage. The data also show that the degranulation type of mast cells and eosinophils in this study is mainly piecemeal. It indicates that the nature of the degranulation type belongs to a chronic process [37]. The local inflammation may progress to chronic status in the tissue without any further medical intervention.

Striking epithelial damage of colonic mucosa of the sensitized mice after challenge with OVA was observed in this study. This phenomenon has been well-documented in airway allergy [38]. Although ulcers are one of the main clinical signs in inflammatory bowel diseases, the etiology is not clear. There have not been many studies considering an association between the ulcers and allergic reactions in the gastrointestinal tract. Eosinophil released major basic protein is suggested to be the major offender to cause epithelial cell exfoliation in the airway mucosa [38]. The erosion and prominent damage to the epithelial barrier can explain the phenomenon of bacterial adhering to and penetrating to colonic mucosa in this study. These bacteria are commensal bacteria. They usually are not considered pathogenic. The damaged epithelium may provide an entry port for the colonizing bacteria to invade the colonic mucosa and to establish an infection.

## Conclusion

In summary, we reported a murine model of ulcerative colitis in this paper that was induced with sinusitis-derived SEB and OVA sensitization followed by repeat challenges with specific antigen OVA. The histopathology of the colonic mucosa included inflammatory cell infiltration, activation of mast cell/eosinophil, epithelial barrier damage, micro-ulcer formation on the surface of colonic mucosa, bacteria translocation and abscesses formation in the subepithelial region.

## List of abbreviations

UC, ulcerative colitis; CS, chronic sinusitis; FESS: functional endoscopic sinus surgery; OVA, ovalbumin; SEB, Staphylococcus enterotoxin B; MPO, myeloperoxidase; HRP, horseradish peroxidase.

## Competing interests

The author(s) declare that they have no competing interests.

## Authors' contributions

PCY was involved in study design, histology, EM observation, data analysis and manuscript preparation; CSW and AZY were involved in SWF collection and animal model.

## Pre-publication history

The pre-publication history for this paper can be accessed here:


