# Correction: a murine model of ulcerative colitis: induced with sinusitis-derived superantigen and food allergen. BMC Gastroenterol. 2005, 5:6

**DOI:** 10.1186/1471-230X-6-23

**Published:** 2006-08-09

**Authors:** Ping-Chang Yang, Chang-Sheng Wang, Zi-Yuan An

**Affiliations:** 1 Department of Pathology and Molecular Medicine, McMaster University, Hamilton, Ontario, Canada; 2 Department of Otolaryngology, Shanxi Medical University, the First Hospital, Taiyuan, Shanxi, China; 3 Division of Gastroenterology, Department of Internal Medicine, Shanxi Medical University, the First Hospital, Taiyuan, Shanxi, China

## 

We used an inappropriate electron photomicrograph in our published paper [1]. The Figure 6C should be replaced with the electron photomicrograph below (C). Figure legend for this picture is not changed.

**Figure 6:**
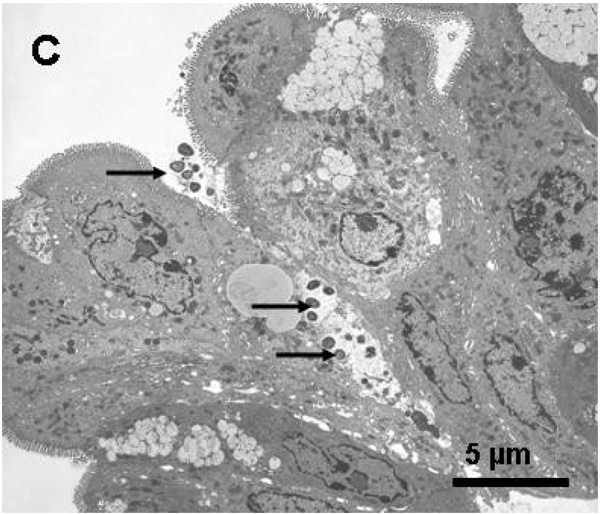
**Ultrapathology of the colonic mucosa of the sensitized mice after challenge with OVA**. Representative EM photomicrographs are taken from the colonic mucosa of the sensitized mice after challenge with OVA and show (C) bacteria (arrows) adhering to and penetrating the epithelial cells (×3,000)

## Pre-publication history

The pre-publication history for this paper can be accessed here:

http://www.biomedcentral.com/1471-230X/6/23/prepub
